# ELK-1 ubiquitination status and transcriptional activity are modulated independently of F-Box protein FBXO25

**DOI:** 10.1074/jbc.RA120.014616

**Published:** 2020-12-24

**Authors:** Reyna Sara Quintero-Barceinas, Franziska Gehringer, Charles Ducker, Janice Saxton, Peter E. Shaw

**Affiliations:** Transcription and Signal Transduction Lab, School of Life Sciences, Queen’s Medical Centre, University of Nottingham, Nottingham, UK

**Keywords:** gene regulation, ETS transcription factor family, ubiquitin ligase, cell proliferation, development, ubiquitin-specific protease 17 (USP17), bHLH, basic helix–loop–helix protein, CHIP, C-terminus of HSP70 interacting protein, CHX, cycloheximide, DMEM, Dulbecco’s Modified Eagle’s Medium, ERKs, extracellular signal-regulated kinases, FCS, fetal calf serum, IMAC, immobilized metal affinity chromatography, MEFs, murine embryonic fibroblasts, MRTFs, myocardin-related transcription factors, mUBQ, monoubiquitin, pUBQ, polyubiquitin chains, qRT-PCR, quantitative reverse transcription–polymerase chain reaction, SRF, serum response factor, TPA, tetradecanoylphorbol acetate, USP17, ubiquitin-specific protease 17, UPS, ubiquitin-proteasome-system

## Abstract

The mitogen-responsive, ETS-domain transcription factor ELK-1 stimulates the expression of immediate early genes at the onset of the cell cycle and participates in early developmental programming. ELK-1 is subject to multiple levels of posttranslational control, including phosphorylation, SUMOylation, and ubiquitination. Recently, removal of monoubiquitin from the ELK-1 ETS domain by the Ubiquitin Specific Protease USP17 was shown to augment ELK-1 transcriptional activity and promote cell proliferation. Here we have used coimmunoprecipitation experiments, protein turnover and ubiquitination assays, RNA-interference and gene expression analyses to examine the possibility that USP17 acts antagonistically with the F-box protein FBXO25, an E3 ubiquitin ligase previously shown to promote ELK-1 ubiquitination and degradation. Our data confirm that FBXO25 and ELK-1 interact in HEK293T cells and that FBXO25 is active toward Hand1 and HAX1, two of its other candidate substrates. However, our data indicate that FBXO25 neither promotes ubiquitination of ELK-1 nor impacts on its transcriptional activity and suggest that an E3 ubiquitin ligase other than FBXO25 regulates ELK-1 ubiquitination and function.

In multicellular organisms, the importance of restricting cell proliferation is such that mitogen-responsive transcription factors are subject to multiple levels of control. For example, the ETS transcription factor ELK-1 is phosphorylated by extracellular signal-regulated kinases (ERKs) in response to growth factor signaling and transiently activated to initiate a gene expression program for cell cycle onset and proliferation (1, 2, 3). Activation of a key subset of target genes is dependent on recruitment of ELK-1 to their promoters in a ternary complex with serum response factor (SRF) (4, 5). Conversely, ELK-1 binding has also been correlated with polycomb repressor complexes in chromatin (6, 7) and in this context appears able to repress developmental gene expression. Furthermore, ELK-1 can compete with myocardin-related transcription factors (MRTFs) for binding to SRF at muscle-specific gene promoters (8, 9, 10). Notably, in amphibian and primitive chordate embryos, inactivation of the gene for ELK-1 is associated with defects that include the precocious appearance of mesoderm, suggesting that ELK-1 participates in the suppression of mesoderm formation (11, 12). Thus ELK-1 contributes to the coordination of cell proliferation with tissue specification and serves as a paradigm for mitogen-responsive transcription factors. Understanding how ELK-1 is regulated is therefore an important goal in cancer and developmental biology.

Aside from phosphorylation, other signaling axes that impinge on ELK-1 within the ERK/ELK-1/SRF regulatory nexus are SUMOylation, which represses ELK-1 transcriptional activity (6, 7), and the ubiquitin proteasome system (UPS), which regulates ELK-1 turnover (13). Moreover, ELK-1 is reversibly monoubiquitinated, which interferes with its activity, and the cell cycle regulated ubiquitin-specific protease USP17 is able to deubiquitinate and reactivate ELK-1 (14, 15, 16). Depletion of USP17 slowed cell proliferation but expression of an ELK-1 mutant refractory to monoubiquitination partially rescued the proliferation defect. USP17 knockdown also caused heterogeneous differentiation of murine ES cells (17). Thus, reversible ELK-1 monoubiquitination may play a pivotal role in coordinating cell proliferation during early development, and it follows that the E3 ubiquitin ligase responsible for monoubiquitination of ELK-1 is central to this regulatory mechanism.

FBXO25 is one of a number of F-box proteins that serve as substrate recognition subunits for SKP1/Cullin1/F-box protein (SCF) complexes, a subfamily of E3 ubiquitin ligases (18). It is closely related to Atrogin-1/FBXO32, an E3 ligase expressed in striated muscle and associated with muscle atrophy (19). Whereas FBXO32 expression is muscle-specific, FBXO25 is expressed in neuronal cells and the human *FBXO25* gene is disrupted in a patient with intellectual disability and epileptic seizures (20). FBXO25 was recently identified as an E3 ubiquitin ligase that promotes the ubiquitination and degradation of ELK-1 (21, 22). We therefore examined whether FBXO25 could also catalyze the monoubiquitination of ELK-1 and thereby operate in opposition to USP17. Although we confirmed that FBXO25 can interact with ELK-1 in cells, an antagonistic relationship between FBXO25 and USP17 toward ELK-1 was not apparent. Indeed, although FBXO25 activity was confirmed with two other candidate substrates, we were unable to detect ubiquitination of ELK-1 or modulation of its function by FBXO25, as previously reported (22).

## Results and discussion

ELK-1 is a substrate for both mono- and polyubiquitination, and we recently showed that USP17 removes monoubiquitin (mUBQ) from ELK-1 (13, 14). USP17 also removes polyubiquitin chains (pUBQ) from ELK-1 (see below), implying an antagonistic relationship between USP17 and FBXO25, an E3 ubiquitin ligase recently reported to catalyze polyubiquitination of ELK-1 (22).

**Figure 1:**
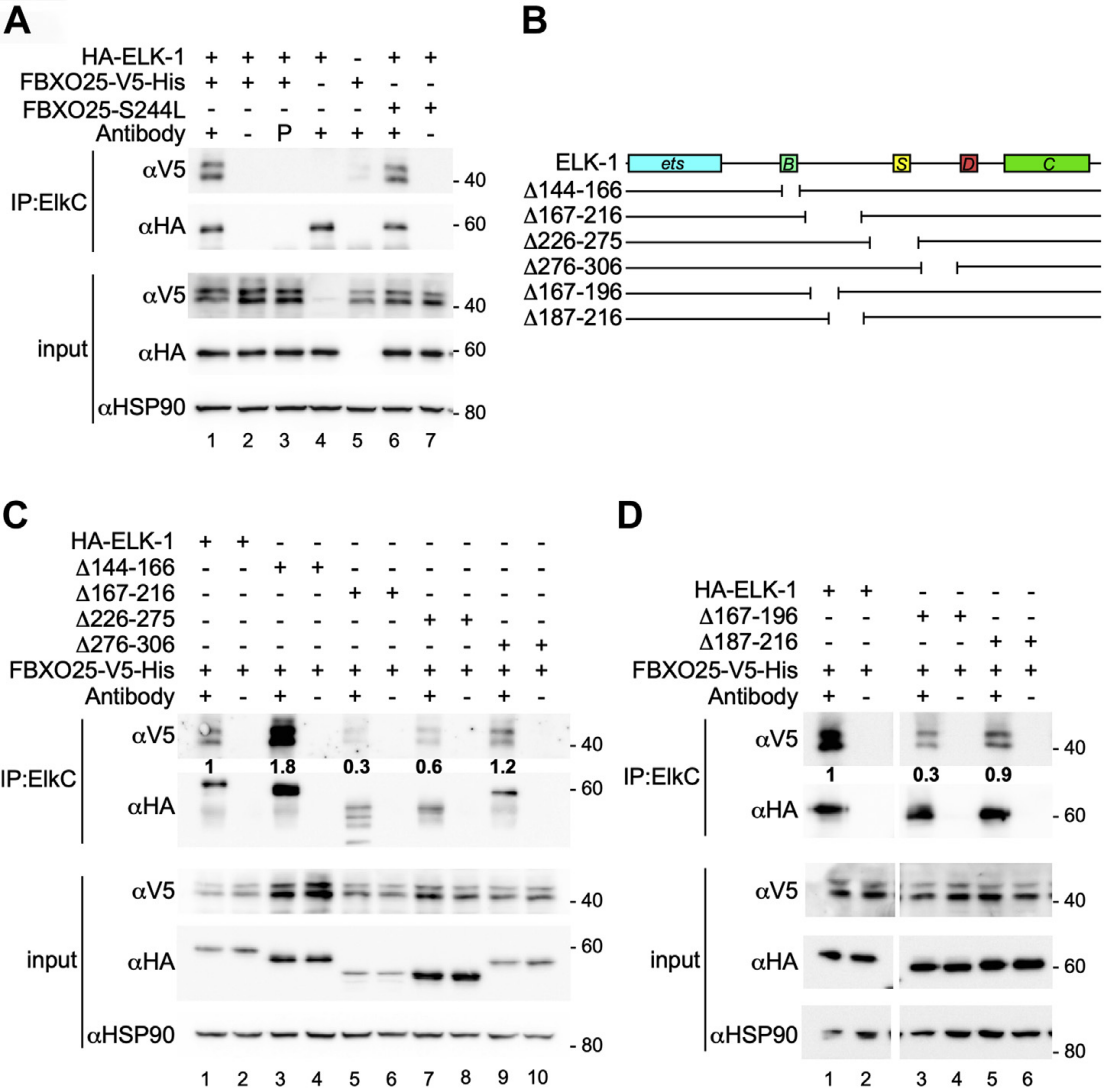
**FBXO25 can interact with ELK-1 *via* cryptic degron sequences.***A*, HEK293T cells were transfected with expression vectors for ELK-1 and FBXO25 or FBXO25-S244L, as indicated. ELK-1 was recovered from lysates by immunoprecipitation (IP) with polyclonal αElkC antibody (P indicates preimmune control), precipitated proteins were resolved by SDS-PAGE and immunoblotted with the antibodies indicated; FBXO25/FBXO25-S244L was detected with αV5 antibody. The experiment was performed three times with similar results. *B*, schematic representation of ELK-1 and its deletion mutants used in Co-IP experiments showing domain structure of ELK-1, consisting of N-terminal ETS domain; SRF-interaction domain (B-box); SUMO-regulated inhibitory domain, mitogen-activated protein kinase (MAPK) docking motif (D-box); C-terminal transactivation domain. Sequences absent from the deletion mutants are indicated below. *C*, HEK293T cells were transfected with expression vectors for FBXO25 and ELK-1 or the ELK-1 deletion mutants indicated. Co-IP experiments were performed as described in part (*A*). Numbers between the first and second panels represent the FBXO25/ELK-1 deletion mutant ratio in relation to FBXO25/ELK-1 ratio. The experiment was performed four times with similar results. *D*, cells were transfected with FBXO25 and ELK-1 or ELK-1 deletion mutants Δ167 to 196 or Δ187 to 216, as indicated. Co-IP experiments were performed as described in part (*A*). Panels on left and right are from the same blots; irrelevant bands in between were cut out. Numbers between the first and second panels represent the FBXO25/ELK-1 deletion mutant ratio in relation to FBXO25/ELK-1 ratio. The experiment was performed three times with similar results.

**Figure 2:**
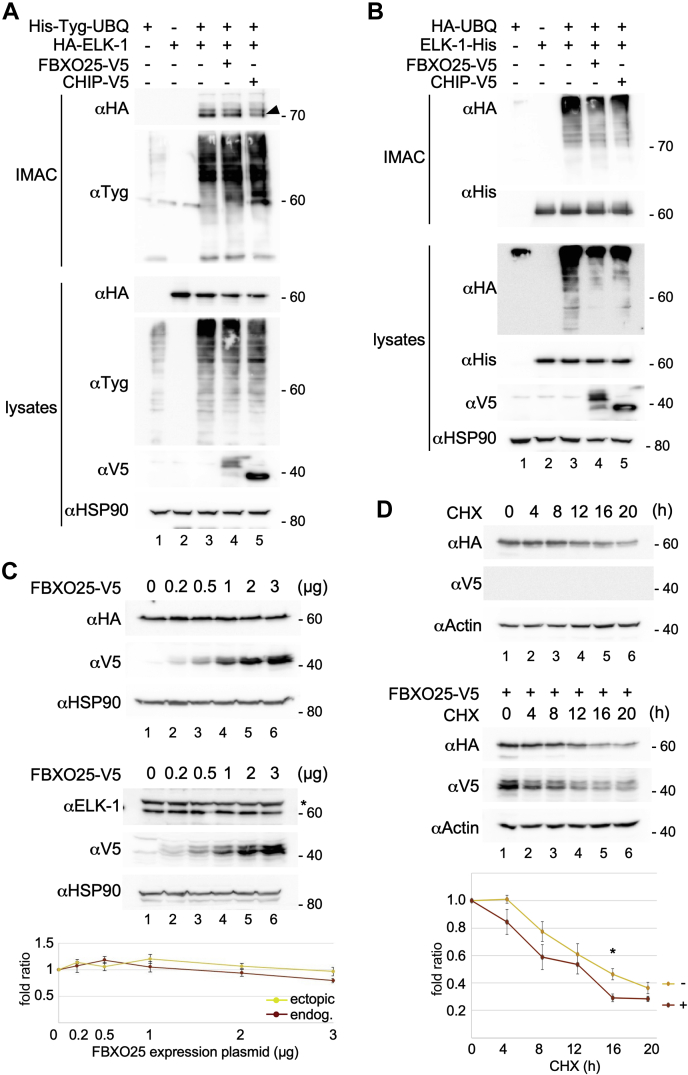
**FBXO25 has no effect on ELK-1 ubiquitination or stability.***A*, HEK293T cells were cotransfected with expression plasmids for His-Tyg-Ubiquitin, HA-ELK-1, FBXO25-V5, CHIP-V5, or empty vector as indicated. Whole-cell lysates were prepared and 10% was used as input control; from the remainder, His-Tyg-Ubiquitin conjugates were purified by IMAC under denaturing conditions. IMAC and lysate fractions were analyzed by SDS-PAGE and immunoblotting using the antibodies indicated. NB, the Tyg epitope is EVHTNQDPLD. Arrowhead indicates ELK-mUBQ. Higher MW species are a mixture of di/triubiquitinated and multiply monoubiquitinated ELK-1 (14, 15). The experiment was performed twice with the same result. *B*, HEK293T cells were cotransfected with expression plasmids for ELK-1-His, HA-UBQ FBXO25-V5, CHIP-V5, or empty vector as indicated. Whole-cell lysates were prepared and 10% was used as input control; from the remainder, His-tagged ELK-1 was purified by IMAC under denaturing conditions. IMAC and lysate fractions were analyzed by SDS-PAGE and immunoblotting using the antibodies indicated. The experiment was performed twice with the same result. C, *Upper panel*, an expression plasmid for HA-ELK-1 was transfected alone (1 μg) or together with increasing amounts (0.2, 0.5, 1, 2, 3 μg) of FBXO25 plasmid, supplemented with empty vector for constant DNA input, into HEK293T cells. Cells were lysed and equal amounts of protein were assayed by SDS-PAGE and immunoblotting. Levels of HA-ELK-1 or FBXO25-V5 were detected using αHA and αV5 antibodies, respectively. HSP90 was used as loading control. *Lower panel*, increasing amounts (0.2, 0.5, 1, 2, 3 μg) of FBXO25 plasmid, supplemented with empty vector for constant DNA input, were transfected into HEK293T cells. Cells were lysed and equal amounts of protein were assayed by SDS-PAGE and immunoblotting. The levels of endogenous ELK-1 (lower band; *asterisk* indicates nonspecific signal) or overexpressed FBXO25-V5 were detected using H160 and αV5 antibodies respectively. HSP90 was used as loading control. *Lower panel* shows densitometric quantification of the results, in which levels of ELK-1 were normalized to HSP90. For all experiments n = 3. *D*, HEK293T were transfected with expression plasmid for HA-ELK-1 and either empty vector (LH panel) or expression vector for FBXO25-V5 (RH panel). After 40 h cells were treated with 200 μM CHX for 0, 4, 8, 12, 16, and 20 h. Cell were lysed and equal amounts of protein were resolved by SDS-PAGE. Immunoblot analysis was performed with the antibodies indicated. Lower panel shows densitometric quantification of the results using AIDA Image Analyzer software. ELK-1 levels were normalized to actin. For all experiments n = 3. Significance was evaluated by Student’s *t*-test. A significant difference in turnover was detected only at the 16-h time point (*p* = 0.047).

ELK-1 was first identified as a potential substrate for FBXO25 through *in vitro* interactions (21). Using a V5/His-tagged version of FBXO25, we could detect its interaction with ELK-1 in HEK293T cells [Fig. 1*A*]. FBXO25 with an S244L mutation in the F-box thought to impair interactions with SKP1 also interacted with ELK-1 (20). We used ELK-1 deletions to map the surface on ELK-1 required for interaction with FBXO25. ELK-1 deletions Δ167 to 216 and Δ167 to 196 [see Fig. 2*B*] localize the interaction with FBXO25 to a region encompassing a cryptic degron in ELK-1 that was defined previously (13) [Fig. 1, *C*–*D*]. These results confirm that ectopically expressed FBXO25 and ELK-1 interact in cells (22).

Expression of FBXO25 in HEK293T cells was reported to increase ELK-pUBQ levels (22). To determine if FBXO25 catalyzed monoubiquitination of ELK-1, we used immobilized metal affinity chromatography (IMAC) to isolate his-tagged UBQ-ELK-1 conjugates from denatured HEK293T cell lysates. However, ectopic expression of V5-tagged FBXO25 (without His-tag) had no more effect on levels of monoubiquitinated ELK-1 (ELK-mUBQ) than the C terminus of HSP70 interacting protein (CHIP), which we used as a negative control [Fig. 2*A*]. Moreover, ubiquitination assays also revealed no effect of FBXO25 (or CHIP) expression on levels of polyubiquitinated ELK-1 (ELK-pUBQ) [Fig. 2*B*]. Since cell lysis and IMAC were performed under denaturing conditions, associations between ELK-1 and noncovalently bound pUBQ chains are ruled out.

Ectopic expression of FBXO25 in HEK293T cells was reported to reduce steady-state levels of ELK-1 and to increase the rate of ELK-1 turnover (22). However, we observed that steady-state levels of endogenous or ectopically expressed ELK-1 were unaffected by FBXO25 expression [Fig. 2*C*]. Furthermore, cycloheximide chase experiments suggested that FBXO25 expression only marginally affected the half-life of ELK-1 [Fig. 2*D*].

**Figure 3:**
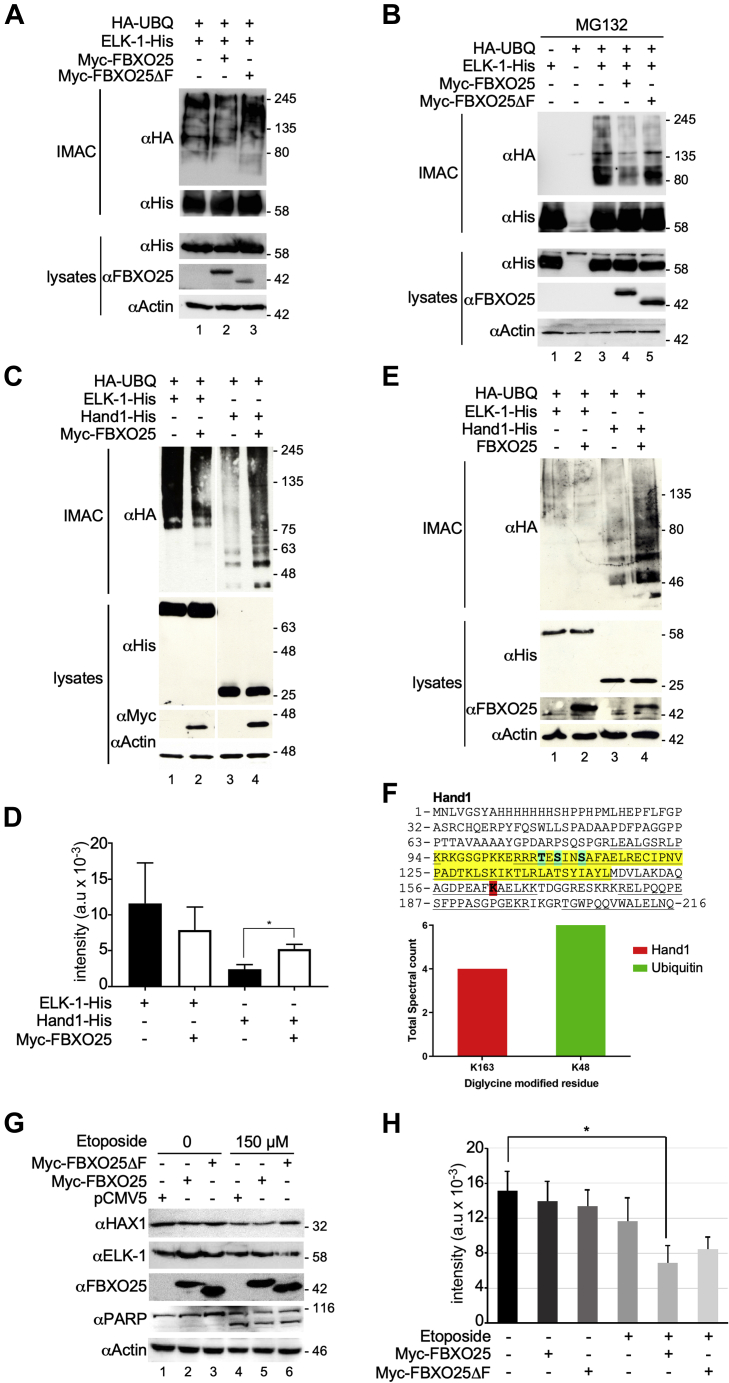
**FBXO25 affects Hand1 ubiquitination and HAX1 stability in apoptotic cells.***A*, whole-cell extracts from HEK293T cells transfected with expression vectors for ELK-1-His, HA-UBQ, Myc-FBXO25, and Myc-FBXO25ΔF, as indicated, were subjected to IMAC under denaturing conditions. Isolated proteins were analyzed by SDS-PAGE and immunoblotting with the antibodies indicated. The result is representative of three independent experiments. *B*, HEK293T cells transfected with expression vectors for ELK-1-His, HA-UBQ, Myc-FBXO25, and Myc-FBXO25ΔF, as indicated, were treated with MG132 (20 μM) for 6 h, then whole-cell extracts were prepared and subjected to IMAC under denaturing conditions. Isolated proteins were analyzed by SDS-PAGE and immunoblotting with the antibodies indicated. The result is representative of three independent experiments. *C*, whole-cell extracts were prepared from HEK293T cells transfected with expression vectors for ELK-1-His, Hand1-His, HA-UBQ, and Myc-FBXO25, as indicated, and subjected to IMAC under denaturing conditions. Isolated proteins were separated by gradient SDS-PAGE and analyzed by immunoblotting with the antibodies indicated. *D*, densitometric quantification of ELK-1 and Hand1 ubiquitination as a function of FBXO25 expression, as shown in (*C*) (n = 3: ∗*p* < 0.05 [0.0395], Student’s *t*-test). *E*, whole-cell extracts were prepared from HeLa cells transfected with expression vectors for ELK-1-His, Hand1-His, HA-UBQ, and FBXO25, as indicated, and subjected to IMAC under denaturing conditions. Isolated proteins were separated by gradient SDS-PAGE and analyzed by immunoblotting with the antibodies indicated. This experiment was performed twice. *F*, amino acid sequence of murine Hand1 (Q64279.1) with bHLH domain highlighted in *yellow*, K163 in *red* and phosphorylation sites T107, S109, and S112 in *blue*. Underscores denote peptide sequence coverage. The histogram below indicates total spectral counts for K163-containing Hand1 peptides and K48-containing ubiquitin peptides with diglycine remnants (+114). The data are from two experiments. *G*, MEFs were transfected with vectors for FBXO25 and FBXO25ΔF or vector control as indicated. Cells were untreated or treated 48 h posttransfection with etoposide for 24 h as indicated. Levels of endogenous HAX-1, ELK-1, PARP cleavage and ectopically expressed FBXO25 were determined by SDS-PAGE and immunoblotting. *H*, quantification of HAX-1 protein levels (n = 3; ∗*p* < 0.05 [0.034], Student’s *t*-test).

We considered the possibility that the V5 epitope on the C terminus of FBXO25 might interfere with its activity toward ELK-1. We therefore produced N-terminal Myc-tagged versions, corresponding to *FBXO25* mRNA variants 1 and 2 (NM_183421 and NM_183420), as well as a mutant version lacking the F-box domain (amino acids 228–266; ΔF) that cannot interact with SKP1 or other components of the SCF1 complex. Consistent with the results obtained using FBXO25-V5, no increase in ELK-pUBQ was observed upon coexpression of Myc-FBXO25 in HEK293T cells, with or without treatment with MG132 [Fig. 3, *A*–*B*].

To confirm the functional integrity of ectopically expressed FBXO25, we turned to other potential client proteins. FBXO25 was reported to ubiquitinate several cardiac-specific transcription factors including Hand1, a basic Helix-Loop-Helix (bHLH) protein (23). Myc-FBXO25 expression significantly increased levels of polyubiquitinated Hand1 in HEK293T cells [Fig. 3, *C*–*D*]. We also assessed the activity of FBXO25 in HeLa cells and found that its expression increased levels of polyubiquitinated Hand1 but did not promote polyubiquitination of ELK-1 [Fig. 3*E*] (see also Supplementary Information). Diglycine remnant mapping of UBQ-Hand1 conjugates from HEK293T cells using protein mass spectrometry confirmed ubiquitination of Hand1 on lysine 163, which maps C terminal to the bHLH domain [Fig. 3*F*] (see also Supplementary Information).

FBXO25 has also been shown to target the HCLS1-associated protein X-1 (HAX1) after apoptotic stress (24). We found that Myc-FBXO25 expression in MEFs accelerated the loss of HAX1 induced by etoposide, as previously reported (24), whereas the ΔF-box mutant did not [Fig. 3, *G*–*H*]. No corresponding change in the level of endogenous ELK-1 was observed. On the basis of the results with Hand1 and HAX-1, we conclude that ectopically expressed FBXO25 is active in cells, although its activity appears to be modest.

**Figure 4:**
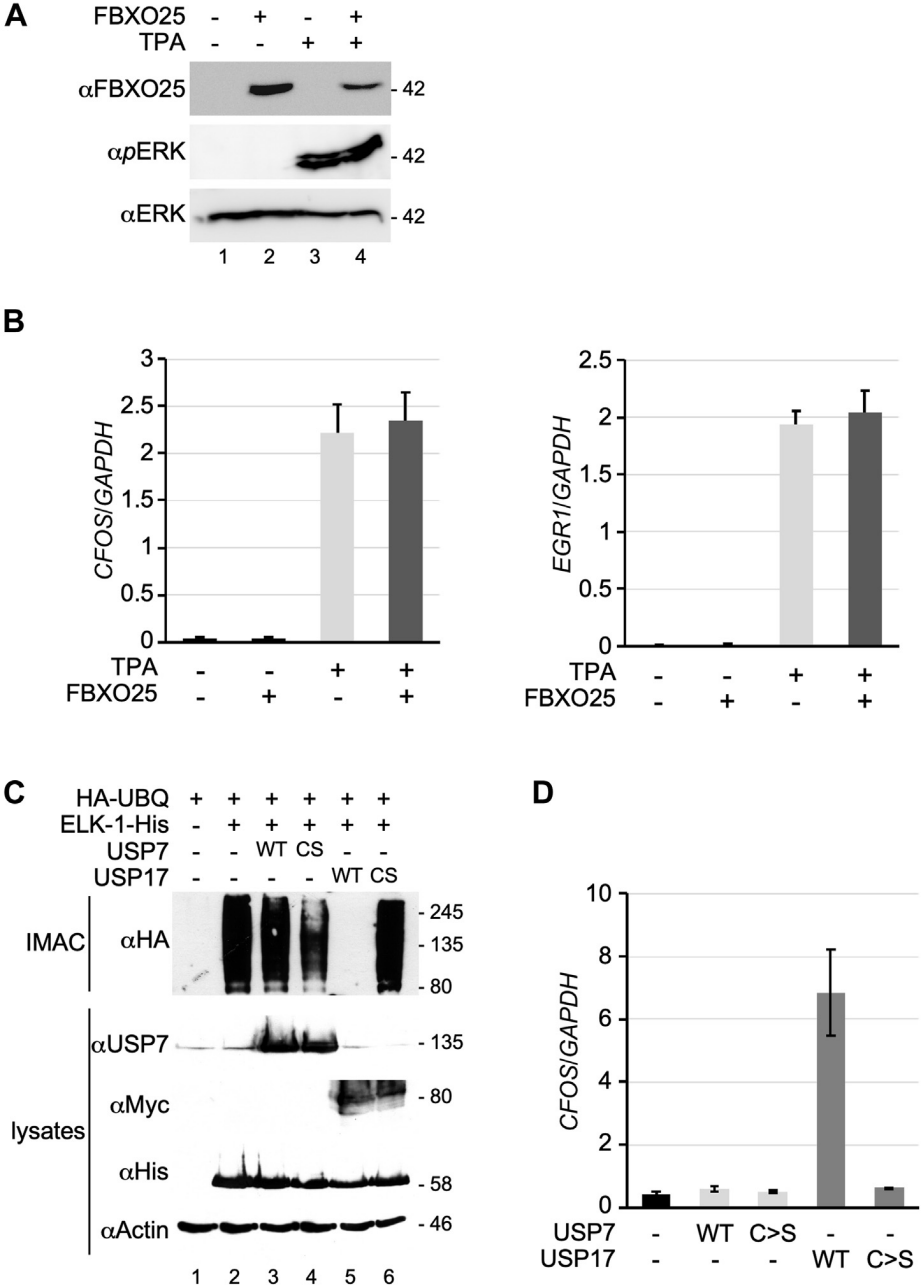
**USP17 but not FBXO25 modulates ELK-1 ubiquitination and target genes.***A*, HEK293T cells were transfected with expression vector for FBXO25 or vector control as indicated. After 48 h cells were untreated or stimulated with tetradecanoylphorbol acetate (TPA) for 1 h as indicated. Whole-cell extracts were prepared and analyzed by SDS-PAGE and immunoblotting for FBXO25 expression and ERK phosphorylation as indicated. *B*, RNA was prepared from cells treated as in (*A*) and *CFOS* and *EGR1* mRNA were analyzed by qRT-PCR. Levels are presented as fold ratios to *GAPDH* (n = 3). Error bars show SEM. *C*, HEK293T cells were transfected with expression vectors for ELK-1-His, HA-UBQ, USP7 (WT and C > S mutant), or Myc-USP17 (WT and C > S mutant), as indicated. Whole-cell extracts were prepared and subjected to IMAC under denaturing conditions. Isolated proteins were separated by gradient SDS-PAGE and analyzed by immunoblotting with the antibodies indicated. This experiment was performed twice. *D*, RNA was prepared from cells treated as in (*C*) and *CFOS* mRNA was analyzed by qRT-PCR. Levels are presented as fold ratios to *GAPDH* (n = 3). Error bars show SEM.

Ectopic FBXO25 expression was previously shown to impair ELK-1 target gene expression in HEK293T cells (22), and conceivably, gene expression may be sensitive to even slight changes in ELK-1 ubiquitination. However, in unstimulated and mitogen-stimulated HEK293T cells, we found that mRNA levels of ELK-1 target genes *CFOS* and *EGR1* were unaffected by FBXO25 expression [Fig. 4, *A*–*B*]. In contrast, expression of USP17, but not a catalytically inactive USP17 mutant or USP7, had a significant impact on ELK-1 target gene expression, consistent with its ability to deubiquitinate ELK-1 [Fig. 4, *C*–*D*] (14, 15).

**Figure 5:**
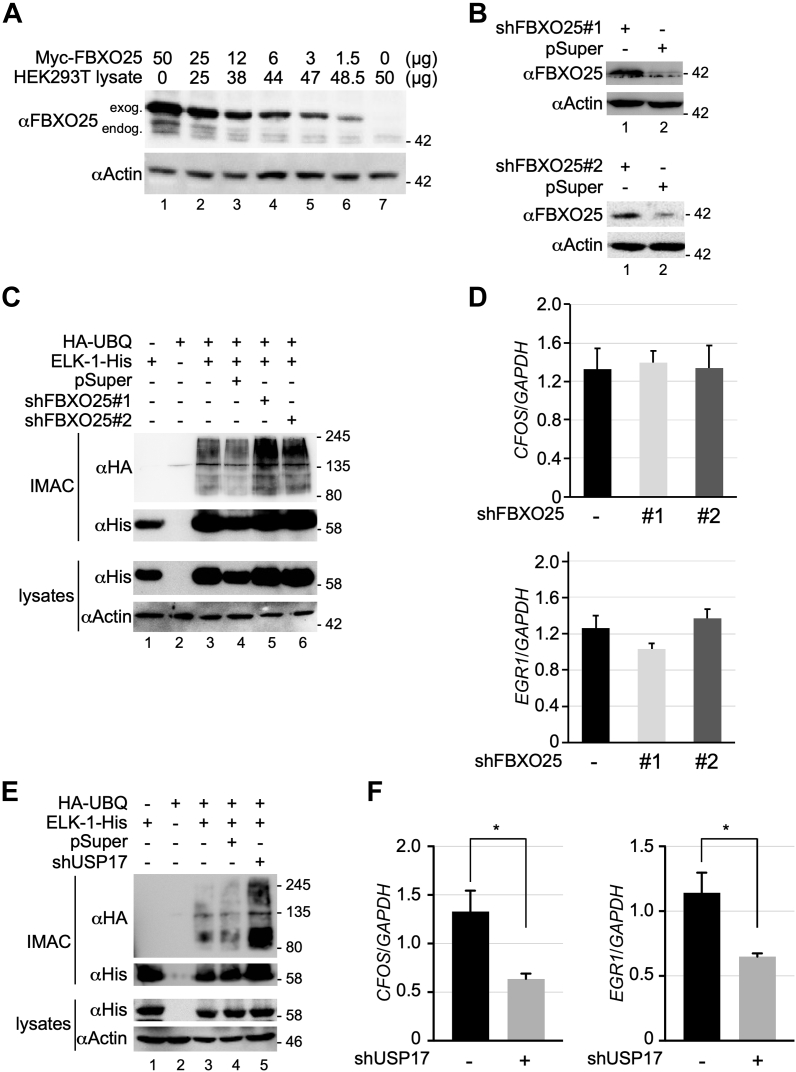
**Depletion of USP17 but not FBXO25 modulates ELK-1 ubiquitination and target genes.***A*, whole cell lysates were prepared from untransfected HEK293T cells and cells transfected with expression vector for FBXO25. Protein concentrations were determined and lysates were mixed at different ratios to titrate out ectopically expressed FBXO25, as indicated. Proteins mixtures were separated by SDS-PAGE and analyzed by immunoblotting with the antibodies indicated. Signals corresponding to exogenous and endogenous FBXO25 are indicated. *B*, whole-cell lysates were prepared from HEK293T cells transfected with shFBXO25#1 and shFBXO25#2 or vector control, as indicated, and FBXO25 levels were determined by SDS-PAGE and immunoblotting. *C*, whole-cell extracts were prepared from HEK293T cells transfected with expression vectors for ELK-1-His, HA-UBQ, shFBXO25#1, shFBXO25#2, or vector control (pSuper) and subjected to IMAC under denaturing conditions. Isolated proteins were analyzed by SDS-PAGE and immunoblotting with the antibodies indicated. The result is representative of two independent experiments. *D*, RNA was prepared from cells treated as in (*C*) and *CFOS* and *EGR1* mRNA were analyzed by qRT-PCR. Levels are presented as fold ratios to *GAPDH* (n = 4). Error bars show SEM. *E*, whole-cell extracts were prepared from HEK293T cells transfected with expression vectors for ELK-1-His, HA-UBQ, shUSP17, or vector control, as indicated, and subjected to IMAC under denaturing conditions. Isolated proteins were analyzed by SDS-PAGE and immunoblotting with the antibodies indicated. The result is representative of two independent experiments. *F*, RNA was prepared from cells treated as in (*E*) and *CFOS* and *EGR1* mRNA were analyzed by qRT-PCR. Levels are presented as fold ratios to *GAPDH* (n = 4; ∗*p* < 0.05 [both 0.019], Student’s *t*-test).

HEK293T cells were reported to express endogenous FBXO25, for which several isoforms exist (25). We first confirmed expression of FBXO25 isoforms (∼3% of ectopic FBXO25 expression) [Fig. 5*A*; lane 7]. To see if this low endogenous FBXO25 expression accounted for ELK-1 polyubiquitination observed in HEK293T cells, we used two shRNAs to target *FBXO25*. Although each shRNA decreased FBXO25 expression in HEK293T cells [Fig. 5*B*], neither diminished polyubiquitination of ELK-1 [Fig. 5*C*]. As with its overexpression, depletion of FBXO25 had no impact on *CFOS* and *EGR1* mRNA levels [Fig. 5*D*]. In contrast, shRNA-mediated depletion of USP17 caused an increase in the levels of ubiquitinated ELK-1 [Fig. 5*E*] and a significant decrease in *CFOS* and *EGR1* mRNA levels [Fig. 5*F*]. These results confirm that altering the ubiquitination status of ELK-1 affects the expression of its target genes (14).

In summary, FBXO25 and USP17 appear unlikely to act antagonistically toward ELK-1. Although ectopically expressed FBXO25 interacted with ELK-1 and the interaction mapped to a previously defined cryptic degron in ELK-1 (13), it did not increase ELK-mUBQ nor ELK-pUBQ levels in HEK293T or HeLa cells, nor did it affect ELK-1 turnover. Conversely, FBXO25 depletion did not decrease ELK-pUBQ levels in HEK293T cells. Moreover, neither expression nor depletion of FBXO25 altered ELK-1 target gene expression. We confirmed that FBXO25 was active toward Hand1 and HAX1, two independently defined FBXO25 substrates (23, 24), and showed that levels of ELK-1 ubiquitination and target gene expression can be modulated in synchrony by varying the ubiquitin profile of ELK-1 with USP17.

Our findings provide further evidence that ubiquitination modulates ELK-1 activity but do not support the notion that FBXO25 is the principal ELK-1 E3 ubiquitin ligase. Possible reasons why our findings differ from those reported previously for FBXO25 (22) include: the likely divergence of HEK293T cells cultured in separate labs over time, the different FBXO25 expression constructs employed, and the alternative experimental conditions used for ubiquitination assays. Our data also indicate that methods to detect interactions between E3 ubiquitin ligases and prospective clients, either *in vitro* or in cells, should not be considered a reliable basis for inferring productive, functional relationships. We conclude that the E3 ubiquitin ligase with an important role in limiting ELK-1 activity during cell proliferation and vertebrate development remains to be identified.

## Experimental procedures

### Cell culture and extract preparation

HEK293T and HeLa cells were grown in Dulbecco’s Modified Eagle’s Medium (DMEM) supplemented with 10% fetal calf serum (FCS), 2 mM *L*-glutamine, 100 U ml^−1^ penicillin, and 100 μg ml^−1^ streptomycin. Murine embryonic fibroblasts (MEFs) were cultured in DMEM high glucose, supplemented with 10% FCS, 100 U ml^−1^ penicillin, and 100 μg ml^−1^ streptomycin. Whole-cell lysates were prepared in a modified RIPA buffer containing 50 mM Tris HCl pH 7.5, 150 mM NaCl, 1% NP-40, 0.5% deoxycholate, 0.1% SDS.

### Plasmids and DNA transfection

Plasmids and sources are listed in the Supplementary Information. All constructs were verified by DNA sequencing. HEK293T cells at a density of 3 × 10^6^ cells per 10 cm dish were transfected with a total of 10 μg DNA *via* calcium phosphate/DNA coprecipitation. HeLa cells were transfected with TransIT-LT1 reagent (Mirus Bio); MEFs were transfected using the K2 transfection system (Biontex).

### Coimmunoprecipitations

Experiments were performed essentially as described previously (26). Cells were lysed in co-IP buffer containing 25 mM Tris HCl pH 7.5, 150 mM KCl, 10 mM NaF, 1 mM Na_3_VO_4_, 1% NP-40, supplemented with mammalian protease and phosphatase inhibitor cocktails (Sigma Aldrich) for 1 h at 4 °C, and cleared by centrifugation. Lysates were normalized for protein concentration, precleared with protein G sepharose beads and immune complexes were allowed to form over night at 4 °C on a rotating wheel. Antibodies used are listed in Supplementary Information. Immune complexes were collected on protein G Sepharose beads by centrifugation, washed five times in co-IP buffer, and released by boiling in SDS gel-loading buffer.

### Ubiquitination assays

Cells were lysed in buffer containing 6 M Guanidinium-HCl and protein–ubiquitin conjugates were captured by immobilized metal affinity chromatography (IMAC). Ni-NTA Agarose beads (Qiagen) were incubated in cell lysates for 2 h at RT and washed five times in 8 M urea. Proteins conjugates were released from the beads in SDS gel-loading buffer containing 200 mM imidazole, resolved by SDS-PAGE, and detected by immunoblotting.

### Protein turnover assays

HEK293T cells were transfected with vectors for ELK-1 and FBXO25 or control vector. Forty hours posttransfection, cells were treated with the translation inhibitor cycloheximide (CHX) at a concentration of 200 μM for 4 to 20 h. Samples were lysed in RIPA buffer, normalized for protein concentration, and ELK-1 protein levels were analyzed by SDS-PAGE and immunoblotting.

### Protein mass spectrometry

HEK293T cells were transfected with a vector encoding His-tagged Hand1 and harvested after 48 h. IMAC-enriched Hand1 was resolved by SDS-PAGE, excised from the gel, reduced, alkylated, and digested with trypsin. Peptides were submitted to tandem mass spectrometry (MS/MS) on an LTQ-Orbitrap spectrometer with nanoflow liquid chromatography (LC). Identification of peptides was conducted in data-dependent mode, utilizing Scaffold (4.11.1) software to assign peaks using the Mascot (2.7.0) search engine, with raw data files run against the human Uniprot database (2018), cRAP database (2019), and a custom Hand1-His sequence (93,735 proteins, 2 max missed cleavages, precursor ion mass tolerance 20 PPM, fragment ion mass tolerance 0.100 Da, peptide threshold 95% minimum, peptide false discovery rate 0.4%). Carbamidomethyl (+57 on C) was considered a fixed modification, while deamidation (+1 on NQ), oxidation (+16 on M), phosphorylation (+80 on STY), and ubiquitination (+114 on K) were considered as variable modifications.

### Gene expression assays

Quantitative RT-PCR analyses were performed as previously described (26). Cells were homogenized and RNA purified using the Nucleospin RNA Kit (Macherey-Nagel). RNA was checked for quantity and quality using the Nanodrop 2000 (Thermo-Scientific) and RNA Nano BioAnalyzer chip (Agilent Technologies). Total RNA (1000 ng) was enriched for mRNA using Dynabeads mRNA Purification Kit (Ambion by Life Technologies) prior to cDNA synthesis. For cDNA synthesis, 500 ng of total RNA or mRNA eluted from Dynabeads was reverse transcribed using AffinityScript reverse transcriptase (Agilent Technologies) in a total reaction volume of 23 μl. Reactions were incubated at 25 °C for 10 min, 50 °C for 60 min, and 70 °C for 15 min to terminate the reaction. Gene expression was quantified using the relative standard curve method. Primers and probes were designed using Primer Express Software and synthesized by Eurofins Genomics (Germany). Gene-specific primers and probes are listed in the Supplementary Information.

### Densitometry and statistical analysis

Densitometry was performed using Image J or AIDA software. Densitometry and qRT-PCR data are presented as mean ± SEM. Statistical analyses were performed with Student’s *t*-test. Significance is reported in figures by ∗*p* < 0.05, ∗∗*p* < 0.01.

## Data availability

The mass spectrometry proteomics data have been deposited with the ProteomeXchange Consortium *via* the PRIDE partner repository under the data set identifier PXD022407 and 10.6019/PXD022407. Hand1 (and ubiquitin) peptides are tabulated in Supplementary Information. All other relevant data are contained within the article. Further information on plasmids is available upon request.

## Conflict of interest

The authors declare that they have no conflicts of interest with the contents of this article.
