# Supplementary material for: ELK-1 ubiquitination status and transcriptional activity are modulated independently of F-Box protein FBXO25
Source: J Biol Chem. 2020 Dec 24;296:100214. doi: 10.1074/jbc.RA120.014616 (PMC7948486; doi:10.1074/jbc.RA120.014616)

**SUPPLEMENTARY INFORMATION**

**FBXO25 pseudo-substrate ELK-1**

**Barceinas, Gehringer et al**

**Table S1. Antibodies**

| ANTIBODY | CODE | SOURCE |
|---|---|---|
| ELK-1 | H160 (SC-22804) | Santa Cruz |
| ElkC | polyclonal | Shaw Lab |
| ELK-1 | E277 | Abcam |
| FBXO25 | AB57051 | Abcam |
| FBXO25 | c397856 | Life Span Biosciences |
| ERK | C14 (SC-154) | Santa Cruz |
| phospho-ERK | E4 (SC-7383) | Santa Cruz |
| HA | 3F10 (11867423001) | Roche |
| His | MCA1396GA | ADB Serotec |
| Tyg (EVHTNQDPLD)[+] | BB2 | hybridoma |
| V5 | ABJ792 | Millipore |
| Myc | SC-40 | Santa Cruz |
| HAX1 | AF5458 | Novus Biologicals |
| USP17/DUB-3 | PA5-44961 | Invitrogen |
| Actin | A2066 | Sigma |
| PARP | 550429 | BD Biosciences |
| Tubulin | SC-8035 | Santa Cruz |
| HSP90$\alpha/\beta$ | F-8 | Santa Cruz |

[+] Bastin, P., Bagherzadeh, A., Matthews, K.R., Gull, K. A novel epitope tag system to study protein targeting and organelle biogenesis in *Trypanosoma brucei*. Mol Biochem Parasitol. (1996) 77, 235–239

**Table S2. Expression and shRNA plasmids**

| PROTEIN | VECTOR | SOURCE |
|---|---|---|
| FBXO25-V5-His | pcDNA4 | Vera Kalscheuer, Berlin |
| FBXO25-V5-His(S244L) | pcDNA4 | Vera Kalscheuer, Berlin |
| FBXO25-V5 | pcDNA4 | This work |
| FBXO25ΔF-V5 | pcDNA4 | This work |
| FBXO25 (untagged) | pcDNA3.1 | Ursula Baumann, Heidelberg |
| Myc-FBXO25 | pCMV5m | This work |
| Myc-FBXO25DF | pCMV5m | This work |
| CHIP-V5 | pcDNA4 | This work (Joerg Hoehfeld, Bonn) |
| His.ELK-1 | pCMV5 | Gille et al (1995) |
| HA.ELK-1 | pCMV5L | Evans et al (2011) |
| HA.ELK-1(D144-166) | pCMV5L | Evans et al (2011) |
| HA.ELK-1(D167-216) | pCMV5L | Evans et al (2011) |
| HA.ELK-1(D226-275) | pCMV5L | Evans et al (2011) |
| HA.ELK-1(D276-306) | pCMV5L | Evans et al (2011) |
| HA.ELK-1(D167-196) | pCMV5L | Evans et al (2011) |
| HA.ELK-1(D187-216) | pCMV5L | Evans et al (2011) |
| HA.Ubiquitin | pCMV5 | Simon Dawson, Nottingham |
| His.Tyg.Ubiquitin | pCMV5 | Gu Wei, New York |
| His.Hand1 | pCMV5 | This work (Ross Breckenridge) |
| USP7 (WT + C>S) | pcI | Roger Everett, Glasgow |
| USP17 (WT + C>S) | pcDNA3 | Daniele Guardavaccaro, Utrecht |
| pshFBXO25#1 | pSUPER | This work |
| pshFBXO25#2 | pSUPER | This work |
| pshUSP17 | pSUPER | Daniele Guardavaccaro, Utrecht |

**Table S3. shRNA target sequences**

| | | |
|---|---|---|
| pshFBXO25#1 | NM_183421 | GAGAGAATAACCGTTGTAA |
| pshFBXO25#2 | NM_183421 | GACACTAACGGCCAATAAT |
| pshUSP17 | NM_201402.2 | GCAGGAAGATGCCCATGAA |

**Table S4. RT-PCR Probes and Primers**

| Gene | NCBI ID | Forward primer (5'-3') | Reverse primer (5'-3') | Taqman Probe (5'-3') |
|---|---|---|---|---|
| *CFOS* | NM_00525.2 | ACTACCACTCACCCGCAGAC | GTGGGAATGAAGTTGGCACT | CCTGTCAACGCGCAGGACTTCTG |
| *EGR1* | NM_001964.2 | CAGCACCTTCAACCCTCAG | CAGCACCTTCTCGTTGTTCA | CTACGAGCACCTGACCGCAGAGTCTT |
| *GAPDH* | NM_002046.3 | CTGCACCACCAACTGCTTAG | ACAGTCTTCTGGGTGGCAGT | CCCTGGCCAAGGTCATCCATG |

**Table S5. Hand1 peptides**

| Hand1-His | | | | |
|---|---|---|---|---|
| **Sequence** | **Observed M/Z** | **Actual Mass** | **Modifications** | **Mascot Identity** |
| DAQAGDPEAFK | 574.76 | 1147.51 | | 37.5 |
| DAQAGDPEAFK | 574.76 | 1147.51 | | 37.5 |
| DAQAGDPEAFK | 574.76 | 1147.51 | | 37.5 |
| DAQAGDPEAFK | 574.76 | 1147.51 | | 37.5 |
| DAQAGDPEAFK | 383.51 | 1147.51 | | 37.5 |
| DAQAGDPEAFK | 574.76 | 1147.51 | | 37.5 |
| DAQAGDPEAFK | 574.76 | 1147.51 | | 37.5 |
| DAQAGDPEAFK | 574.76 | 1147.51 | | 37.5 |
| DAQAGDPEAFK | 574.76 | 1147.51 | | 37.5 |
| DAQAGDPEAFK | 574.76 | 1147.51 | | 37.5 |
| DAQAGDPEAFK | 574.76 | 1147.51 | | 37.7 |
| DAQAGDPEAFK | 574.76 | 1147.51 | | 37.7 |
| DAQAGDPEAFK | 574.76 | 1147.51 | | 37.5 |
| DAQAGDPEAFK | 574.76 | 1147.51 | | 37.5 |
| DAQAGDPEAFK | 574.77 | 1147.52 | | 37.6 |
| DAQAGDPEAFK | 574.76 | 1147.51 | | 37.5 |
| DAQAGDPEAFK | 574.76 | 1147.51 | | 37.4 |
| DAQAGDPEAFK | 574.76 | 1147.51 | | 37.7 |
| DAQAGDPEAFK | 574.76 | 1147.51 | | 37.5 |
| DAQAGDPEAFK | 574.77 | 1147.52 | | 37.6 |
| DAQAGDPEAFK | 574.76 | 1147.51 | | 37.7 |
| DAQAGDPEAFK | 574.76 | 1147.51 | | 37.7 |
| DAQAGDPEAFK | 574.76 | 1147.51 | | 37.5 |
| DAQAGDPEAFK | 574.76 | 1147.51 | | 37.8 |
| DAQAGDPEAFK | 574.76 | 1147.51 | | 37.5 |
| DAQAGDPEAFK | 574.76 | 1147.51 | | 37.7 |
| DAQAGDPEAFK | 574.76 | 1147.52 | | 37.5 |
| DAQAGDPEAFK | 574.77 | 1147.52 | | 37.5 |
| DAQAGDPEAFK | 574.76 | 1147.51 | | 37.5 |
| DAQAGDPEAFKAELK | 795.39 | 1588.77 | | 40.3 |
| DAQAGDPEAFKAELK | 795.39 | 1588.77 | | 40.3 |
| DAQAGDPEAFKAELK | 530.60 | 1588.77 | | 40.4 |

| | | | | |
|---|---|---|---|---|
| DAQAGDPEAF**K**AELK | 568.61 | 1702.82 | Ubiquitination (+114) | 40.8 |
| DAQAGDPEAFKAELK | 530.60 | 1588.77 | | 40.3 |
| DAQAGDPEAFKAELK | 530.60 | 1588.77 | | 40.3 |
| DAQAGDPEAFKAELK | 795.39 | 1588.77 | | 40.2 |
| DAQAGDPEAFKAELK | 530.60 | 1588.78 | | 40.3 |
| DAQAGDPEAFKAELK | 795.39 | 1588.77 | | 40.4 |
| DAQAGDPEAFKAELK | 530.60 | 1588.77 | | 40.3 |
| DAQAGDPEAF**K**AELK | 852.42 | 1702.82 | Ubiquitination (+114) | 40.7 |
| DAQAGDPEAFKAELK | 530.60 | 1588.77 | | 40.4 |
| DAQAGDPEAFKAELK | 530.60 | 1588.77 | | 40.3 |
| DAQAGDPEAFKAELK | 795.39 | 1588.78 | | 40.3 |
| DAQAGDPEAFKAELK | 530.60 | 1588.77 | | 40.3 |
| DAQAGDPEAFKAELK | 530.60 | 1588.77 | | 40.4 |
| DAQAGDPEAFKAELK | 530.60 | 1588.77 | | 40.3 |
| DAQAGDPEAFKAELK | 530.60 | 1588.77 | | 40.4 |
| DAQAGDPEAFKAELKK | 859.44 | 1716.87 | | 40.1 |
| DAQAGDPEAFKAELKK | 430.22 | 1716.87 | | 40.1 |
| DAQAGDPEAFKAELKK | 430.22 | 1716.87 | | 39.9 |
| DAQAGDPEAFKAELKK | 430.23 | 1716.87 | | 39.9 |
| DAQAGDPEAFKAELKK | 573.30 | 1716.87 | | 40.0 |
| DAQAGDPEAFKAEL**K**K | 458.74 | 1830.92 | Ubiquitination (+114) | 40.7 |
| (R)ECIPNVPADTK(L) | 415.20 | 1242.59 | Carbamidomethyl (+57) | 38.1 |
| (R)ECIPNVPADTK(L) | 622.30 | 1242.59 | Carbamidomethyl (+57) | 38.1 |
| (R)ECIPNVPADTK(L) | 622.30 | 1242.59 | Carbamidomethyl (+57) | 38.1 |
| (R)ECIPNVPADTK(L) | 622.30 | 1242.59 | Carbamidomethyl (+57) | 38.0 |
| (R)ECIPNVPADTK(L) | 622.30 | 1242.59 | Carbamidomethyl (+57) | 38.1 |
| (R)ECIPNVPADTK(L) | 622.30 | 1242.59 | Carbamidomethyl (+57) | 38.1 |
| (R)ECIPNVPADTK(L) | 622.30 | 1242.59 | Carbamidomethyl (+57) | 38.3 |
| (R)ECIPNVPADTK(L) | 622.30 | 1242.59 | Carbamidomethyl (+57) | 37.9 |
| (R)ECIPNVPADTK(L) | 622.30 | 1242.59 | Carbamidomethyl (+57) | 37.9 |
| (R)ECIPNVPADTK(L) | 622.30 | 1242.59 | Carbamidomethyl (+57) | 37.7 |
| (R)ECIPNVPADTK(L) | 622.30 | 1242.59 | Carbamidomethyl (+57) | 38.0 |
| (R)ECIPNVPADTK(L) | 622.30 | 1242.59 | Carbamidomethyl (+57) | 38.1 |
| (R)ECIPNVPADTKLSK(I) | 524.61 | 1570.81 | Carbamidomethyl (+57) | 39.0 |
| (R)ECIP**N**VPADT**K**LSK(I) | 562.96 | 1685.85 | Carbamidomethyl (+57), Deamidated | 39.9 |

| | | | | |
|---|---|---|---|---|
| | | | (+1), Ubiquitination (+114) | |
| ELPQQPESFPPASGPGEK | 632.31 | 1893.91 | | 41.3 |
| ELPQQPESFPPASGPGEK | 947.96 | 1893.91 | | 41.4 |
| ELPQQPESFPPASGPGEK | 947.96 | 1893.91 | | 41.4 |
| ELPQQPESFPPASGPGEK | 947.96 | 1893.91 | | 41.3 |
| ELPQQPESFPPASGPGEK | 947.96 | 1893.91 | | 41.4 |
| ELPQQPESFPPASGPGEK | 947.96 | 1893.91 | | 41.3 |
| ELPQQPESFPPASGPGEK | 632.31 | 1893.91 | | 41.4 |
| ELPQQPESFPPASGPGEK | 947.96 | 1893.91 | | 41.4 |
| ELPQQPESFPPASGPGEK | 947.96 | 1893.91 | | 41.4 |
| ELPQQPESFPPASGPGEK | 947.96 | 1893.91 | | 41.4 |
| ELPQQPESFPPASGPGEK | 947.96 | 1893.91 | | 41.4 |
| ELPQQPESFPPASGPGEK | 947.96 | 1893.91 | | 41.4 |
| ELPQQPESFPPASGPGEK | 947.96 | 1893.91 | | 41.4 |
| ELPQQPESFPPASGPGEK | 632.31 | 1893.91 | | 41.4 |
| ELPQQPESFPPASGPGEK | 947.96 | 1893.91 | | 41.3 |
| ELPQQPESFPPASGPGEK | 947.96 | 1893.91 | | 41.4 |
| ELPQQPESFPPASGPGEK | 947.96 | 1893.91 | | 41.3 |
| ELPQQPESFPPASGPGEK | 947.96 | 1893.91 | | 41.4 |
| ELPQQPESFPPASGPGEK | 947.96 | 1893.91 | | 41.4 |
| ELPQQPESFPPASGPGEK | 947.96 | 1893.91 | | 41.4 |
| ELPQQPESFPPASGPGEK | 947.97 | 1893.92 | | 41.3 |
| ELPQQPESFPPASGPGEK | 632.31 | 1893.91 | | 41.4 |
| ELPQQPESFPPASGPGEK | 632.31 | 1893.91 | | 41.4 |
| ELPQQPESFPPASGPGEK | 947.96 | 1893.91 | | 41.3 |
| ELPQQPESFPPASGPGEK | 947.96 | 1893.91 | | 41.4 |
| ELPQQPESFPPASGPGEK | 632.31 | 1893.91 | | 41.3 |
| ELPQQPESFPPASGPGEK | 947.96 | 1893.91 | | 41.3 |
| ELPQQPESFPPASGPGEK | 632.31 | 1893.91 | | 41.4 |
| ELPQQPESFPPASGPGEK | 947.97 | 1893.92 | | 41.4 |
| ELPQQPESFPPASGPGEK | 947.96 | 1893.91 | | 41.3 |
| ELPQQPESFPPASGPGEK | 947.96 | 1893.91 | | 41.4 |
| ELPQQPESFPPASGPGEK | 947.97 | 1893.92 | | 41.3 |
| ELP**Q**QPESFPPASGPGEK | 948.46 | 1894.91 | Deamidated (+1) | 41.4 |
| ELPQQPE**S**FPPASGPGEK | 987.95 | 1973.88 | Phospho (+80) | 41.5 |
| ELPQQPESFPPASGPGEKR | 684.34 | 2050.01 | | 41.4 |
| ELPQQPESFPPASGPGEKR | 684.34 | 2050.01 | | 41.4 |
| ELPQQPESFPPASGPGEKR | 684.34 | 2050.01 | | 41.4 |
| ELPQQPESFPPASGPGEKR | 684.34 | 2050.01 | | 41.3 |
| ELPQQPESFPPASGPGEKR | 684.35 | 2050.01 | | 41.2 |
| ELPQQPESFPPASGPGE**K**R | 722.36 | 2164.05 | Ubiquitination (+114) | 41.9 |
| (R)LATSYIAYLMDVLAK(D) | 836.45 | 1670.90 | | 38.2 |
| (R)LATSYIAYL**M**DVLAK(D) | 844.45 | 1670.89 | Oxidation (+16) | 38.8 |
| (R)LATSYIAYLMDVLAK(D) | 557.97 | 1670.90 | | 38.2 |
| (R)LATSYIAYLMDVLAK(D) | 836.45 | 1670.90 | | 38.2 |
| (R)LATSYIAYLMDVLAK(D) | 836.45 | 1670.89 | | 38.3 |
| (R)LATSYIAYL**M**DVLAK(D) | 563.30 | 1670.89 | Oxidation (+16) | 38.9 |
| (R)LATSYIAYLMDVLAK(D) | 557.97 | 1670.90 | | 38.0 |
| (R)LATSYIAYL**M**DVLAK(D) | 844.45 | 1670.89 | Oxidation (+16) | 38.6 |
| (R)LATSYIAYL**M**DVLAK(D) | 563.30 | 1670.89 | Oxidation (+16) | 38.6 |
| (R)LATSYIAYLMDVLAK(D) | 836.46 | 1670.90 | | 38.3 |

| | | | | |
|---|---|---|---|---|
| (R)LATSYIAYL**M**DVLAK(D) | 844.45 | 1670.89 | Oxidation (+16) | 38.9 |
| (R)LATSYIAYL**M**DVLAK(D) | 563.30 | 1670.89 | Oxidation (+16) | 38.9 |
| (R)LATSYIAYLMDVLAK(D) | 836.46 | 1670.90 | | 38.0 |
| (R)LATSYIAYLMDVLAK(D) | 557.97 | 1670.89 | | 38.3 |
| (R)LATSYIAYLMDVLAK(D) | 836.46 | 1670.90 | | 38.0 |
| (R)LATSYIAYL**M**DVLAK(D) | 563.30 | 1670.89 | Oxidation (+16) | 38.9 |
| (R)LATSYIAYLMDVLAK(D) | 836.45 | 1670.89 | | 38.6 |
| (R)LATSYIAYLMDVLAK(D) | 836.45 | 1670.89 | | 38.6 |
| (R)LATSYIAYL**M**DVLAK(D) | 844.45 | 1670.89 | Oxidation (+16) | 38.9 |
| LEALGSRLPK | 542.33 | 1082.64 | | 30.4 |
| RELPQQPESFPPASGPGEK | 684.34 | 2050.01 | | 41.4 |
| RELPQQPESFPPASGPGEK | 1026.02 | 2050.02 | | 41.2 |
| RELPQQPESFPPASGPGEK | 1026.02 | 2050.02 | | 41.2 |
| RELPQQPESFPPASGPGEK | 1026.01 | 2050.01 | | 41.3 |
| RELPQ**Q**PESFPPASGPGEK | 684.68 | 2050.01 | Deamidated (+1) | 41.3 |
| (R)RRTESINSAFAELR(E) | 550.63 | 1648.86 | | 38.7 |
| (R)RR**T**ESINSAFAELR(E) | 577.28 | 1728.83 | Phospho (+80) | 40.8 |
| (R)RR**T**ESINSAFAELR(E) | 577.28 | 1728.83 | Phospho (+80) | 40.8 |
| (R)RR**T**ESINSAFAELR(E) | 577.29 | 1728.83 | Phospho (+80) | 40.7 |
| (R)RR**T**ESI**N**SAFAELR(E) | 577.61 | 1729.82 | Phospho (+80), Deamidated (+1) | 40.7 |
| (R)RRTE**S**INSAFAELR(E) | 433.21 | 1728.83 | Phospho (+80) | 40.7 |
| (R)RR**T**ESI**N**SAFAELR(E) | 577.61 | 1729.82 | Phospho (+80), Deamidated (+1) | 40.7 |
| (R)RR**T**E**S**INSAFAELR(E) | 603.94 | 1808.80 | Phospho (+80), Phospho (+80) | 40.8 |
| (R)RR**T**ESINSAFAELR(E) | 865.42 | 1728.83 | Phospho (+80) | 40.8 |
| (R)RRTESINSAFAELR(E) | 413.22 | 1648.86 | | 38.9 |
| (R)RR**T**ESINSAFAELR(E) | 577.28 | 1728.82 | Phospho (+80) | 40.8 |
| (R)R**T**ESINSAFAELR(E) | 525.25 | 1572.73 | Phospho (+80) | 40.1 |
| (R)RTESINSAFAELR(E) | 498.59 | 1492.76 | | 39.1 |
| (R)RTESINSAFAELR(E) | 747.39 | 1492.76 | | 39.1 |
| (R)RTESINSAFAELR(E) | 498.60 | 1492.77 | | 39.0 |
| (R)RTESINSAFAELR(E) | 498.59 | 1492.76 | | 39.1 |
| (R)RTESINSAFAELR(E) | 498.59 | 1492.76 | | 39.1 |
| (R)RTESINSAFAELR(E) | 747.39 | 1492.76 | | 39.1 |
| (R)RTESINSAFAELR(E) | 498.59 | 1492.76 | | 39.1 |
| (R)RTESINSAFAELR(E) | 498.59 | 1492.76 | | 39.1 |
| (R)R**T**ESINSAFAELR(E) | 787.37 | 1572.73 | Phospho (+80) | 40.1 |
| (R)RTESINSAFAELR(E) | 747.39 | 1492.76 | | 39.2 |
| (R)RTESINSAFAELR(E) | 747.39 | 1492.76 | | 39.2 |
| (R)RTESINSAFAELR(E) | 747.39 | 1492.76 | | 39.1 |
| (R)RTE**S**I**N**SAFAELR(E) | 787.86 | 1573.71 | Phospho (+80), Deamidated (+1) | 40.0 |
| (R)RTE**S**IN**S**AFAELR(E) | 551.91 | 1652.69 | Phospho (+80), Phospho (+80) | 39.4 |
| (R)RTESINSAFAELR(E) | 498.60 | 1492.76 | | 39.0 |
| (R)RTESI**N**SAFAELR(E) | 747.89 | 1493.76 | Deamidated (+1) | 39.4 |
| (R)TESINSAFAELR(E) | 446.56 | 1366.66 | | 39.5 |
| (R)TESINSAFAELR(E) | 669.34 | 1366.66 | | 39.4 |
| (R)TESINSAFAELR(E) | 669.34 | 1366.66 | | 39.4 |
| (R)TESINSAFAELR(E) | 669.34 | 1366.66 | | 39.4 |
| (R)TESINSAFAELR(E) | 669.34 | 1366.66 | | 39.4 |

| | | | | |
|---|---|---|---|---|
| (R)TESINSAFAELR(E) | 669.34 | 1366.66 | | 39.4 |
| (R)TESINSAFAELR(E) | 669.34 | 1366.66 | | 39.5 |
| (R)TESINSAFAELR(E) | 669.34 | 1366.66 | | 39.5 |
| (R)**T**ESI**N**SAFAELR(E) | 709.81 | 1417.61 | Phospho (+80), Deamidated (+1) | 38.8 |
| (R)TESINSAFAELR(E) | 669.34 | 1366.66 | | 39.4 |
| (R)TESINSAFAELR(E) | 669.34 | 1366.66 | | 39.5 |
| (R)**T**ESINSAFAELR(E) | 709.32 | 1416.63 | Phospho (+80) | 39.1 |
| (R)**T**ESINSAFAELR(E) | 709.32 | 1416.62 | Phospho (+80) | 38.8 |
| (R)**T**ESINSAFAELR(E) | 709.32 | 1416.63 | Phospho (+80) | 39.0 |
| (R)TESINSAFAELR(E) | 669.34 | 1366.66 | | 39.4 |
| (R)TESINSAFAELR(E) | 669.34 | 1366.66 | | 39.4 |
| (R)TESINSAFAELR(E) | 669.34 | 1366.66 | | 39.4 |
| (R)TESINSAFAELR(E) | 669.34 | 1366.66 | | 39.5 |
| (R)TESIN**S**AFAELR(E) | 473.22 | 1416.63 | Phospho (+80) | 39.1 |
| (R)TE**S**IN**S**AFAELR(E) | 749.30 | 1496.59 | Phospho (+80), Phospho (+80) | 37.4 |
| (R)TESINSAFAELR(E) | 669.34 | 1366.66 | | 39.5 |
| (R)TE**S**INSAFAELR(E) | 709.32 | 1416.63 | Phospho (+80) | 39.0 |
| TGWPQQVWALELNQHHHHHH | 499.24 | 2491.18 | | 42.5 |
| TGWPQQVWALELNQHHHHHH | 499.24 | 2491.18 | | 42.5 |
| TGWPQQVWALELNQHHHHHH | 499.24 | 2491.18 | | 42.5 |
| TGWPQQVWALELNQHHHHHH | 499.24 | 2491.18 | | 42.5 |
| TGWPQQVWALELNQHHHHHH | 499.24 | 2491.18 | | 42.5 |
| TGWPQQVWALELNQHHHHHH | 499.24 | 2491.18 | | 42.5 |
| TGWPQQVWALELNQHHHHHH | 499.24 | 2491.18 | | 42.5 |
| TGWPQQVWALELNQHHHHHH | 499.24 | 2491.18 | | 42.5 |
| TGWPQQVWALELNQHHHHHH | 499.24 | 2491.18 | | 42.5 |
| TGWPQQVWALELNQHHHHHH | 1246.60 | 2491.18 | | 42.5 |
| TGWPQQVWALELNQHHHHHH | 499.24 | 2491.18 | | 42.5 |
| TGWPQQVWALELNQHHHHHH | 499.24 | 2491.18 | | 42.5 |
| TGWPQQVWALELNQHHHHHH | 499.24 | 2491.18 | | 42.5 |
| TGWPQQVWALELNQHHHHHH | 623.80 | 2491.18 | | 42.5 |
| TGWPQQVWALELNQHHHHHH | 499.24 | 2491.18 | | 42.5 |
| TGWPQQVWALELNQHHHHHH | 499.24 | 2491.18 | | 42.5 |
| TGWPQQVWALELNQHHHHHH | 499.24 | 2491.19 | | 42.4 |
| TGWPQQVWALEL**N**QHHHHHH | 624.05 | 2492.18 | Deamidated (+1) | 42.6 |
| TGWPQQVWALELNQHHHHHH | 623.80 | 2491.18 | | 42.6 |
| TGWPQQVWALELNQHHHHHH | 499.24 | 2491.18 | | 42.5 |
| TGWPQQVWALELNQHHHHHH | 623.80 | 2491.18 | | 42.5 |
| TGWPQQVWALELNQHHHHHH | 499.25 | 2491.19 | | 42.5 |
| TGWPQQVWALELNQHHHHHH | 623.80 | 2491.18 | | 42.5 |
| TGWPQQVWALELNQHHHHHH | 499.24 | 2491.18 | | 42.5 |
| TGWPQQVWALELNQHHHHHH | 623.80 | 2491.18 | | 42.5 |
| TGWPQQVWALEL**N**QHHHHHH | 499.44 | 2492.17 | Deamidated (+1) | 42.7 |
| TGWPQQVWALELNQHHHHHH | 623.80 | 2491.18 | | 42.5 |
| TGWPQQVWALELNQHHHHHH | 499.24 | 2491.18 | | 42.5 |
| TGWPQQVWALELNQHHHHHH | 623.80 | 2491.18 | | 42.5 |
| TGWPQQVWALELNQHHHHHH | 623.80 | 2491.18 | | 42.5 |
| TGWPQQVWALELNQHHHHHH | 623.80 | 2491.18 | | 42.5 |
| TGWPQQVWALELNQHHHHHH | 623.80 | 2491.18 | | 42.5 |
| TGWPQQVWALELNQHHHHHH | 623.80 | 2491.18 | | 42.5 |
| TGWPQQVWALELNQHHHHHH | 831.40 | 2491.18 | | 42.5 |

| | | | | |
|---|---|---|---|---|
| TGWPQQVWALELNQHHHHHH | 499.24 | 2491.18 | | 42.5 |
| TGWPQQVWALELNQHHHHHH | 623.80 | 2491.18 | | 42.5 |
| TGWPQQVWALELNQHHHHHH | 623.80 | 2491.18 | | 42.5 |
| TGWPQQVWALEL**N**QHHHHHH | 831.73 | 2492.16 | Deamidated (+1) | 42.7 |
| TGWPQQVWALELNQHHHHHH | 623.80 | 2491.18 | | 42.5 |
| TGWPQQVWALELNQHHHHHH | 623.80 | 2491.18 | | 42.5 |
| TGWPQQVWALELNQHHHHHH | 623.80 | 2491.19 | | 42.5 |
| TGWPQQVWALELNQHHHHHH | 1246.60 | 2491.18 | | 42.5 |
| TGWPQQVWALELNQHHHHHH | 623.80 | 2491.18 | | 42.5 |
| TGWPQQVWALELNQHHHHHH | 831.40 | 2491.18 | | 42.5 |
| TGWPQQVWALELNQHHHHHH | 623.80 | 2491.18 | | 42.5 |
| TGWPQQVWALELNQHHHHHH | 623.80 | 2491.18 | | 42.5 |
| TGWPQQVWALELNQHHHHHH | 623.80 | 2491.18 | | 42.5 |
| TGWPQQVWALELNQHHHHHH | 623.80 | 2491.18 | | 42.5 |
| TGWPQQVWALELNQHHHHHH | 1246.59 | 2491.17 | | 42.6 |
| TGWPQQVWALELNQHHHHHH | 1246.60 | 2491.19 | | 42.5 |
| TGWPQQVWALELNQHHHHHH | 623.80 | 2491.18 | | 42.6 |
| TGWPQQVWALELNQHHHHHH | 831.40 | 2491.18 | | 42.5 |
| TGWPQQVWALELNQHHHHHH | 831.40 | 2491.18 | | 42.5 |
| TGWPQQVWALELNQHHHHHH | 831.40 | 2491.18 | | 42.5 |
| TGWPQQVWALELNQHHHHHH | 623.80 | 2491.19 | | 42.5 |
| TGWPQ**Q**VWALELNQHHHHHH | 1247.10 | 2492.18 | Deamidated (+1) | 42.6 |
| TGWPQQVWALELNQHHHHHH | 831.40 | 2491.18 | | 42.5 |
| TGWPQQVWALELNQHHHHHH | 831.40 | 2491.18 | | 42.5 |
| TGWPQQVWALELNQHHHHHH | 623.80 | 2491.17 | | 42.6 |
| TGWPQQVWALELNQHHHHHH | 499.25 | 2491.19 | | 42.5 |
| TGWPQQVWALELNQHHHHHH | 831.40 | 2491.18 | | 42.5 |
| TGWPQQVWALELNQHHHHHH | 1246.60 | 2491.18 | | 42.5 |
| TGWPQQVWALELNQHHHHHH | 831.40 | 2491.17 | | 42.6 |
| TGWPQQVWALELNQHHHHHH | 831.40 | 2491.18 | | 42.5 |
| TGWP**QQ**VWALELNQHHHHHH | 499.64 | 2493.18 | Deamidated (+1), Deamidated (+1) | 42.5 |
| TGWPQQVWALELNQHHHHHH | 831.40 | 2491.18 | | 42.5 |
| TGWPQQVWALELN**Q**HHHHHH | 831.73 | 2492.17 | Deamidated (+1) | 42.7 |
| TGWPQQVWALELNQHHHHHH | 831.40 | 2491.18 | | 42.5 |
| TGWPQ**Q**VWALEL**N**QHHHHHH | 1247.59 | 2493.16 | Deamidated (+1), Deamidated (+1) | 42.7 |
| TGWPQQVWALELNQHHHHHH | 831.40 | 2491.17 | | 42.5 |
| **Ubiquitin** | | | | |
| **Sequence** | **Observed M/Z** | **Actual Mass** | **Modifications** | **Mascot Identity** |
| (K)TITLEVEPSDTIENVK(A) | 596.65 | 1786.92 | | 39.9 |
| (K)TITLEVEPSDTIENVK(A) | 894.47 | 1786.92 | | 39.9 |
| (K)IQDKEGIPPDQQR(L) | 508.60 | 1522.77 | | 39.5 |
| (K)IQDKEGIPPDQQR(L) | 508.60 | 1522.77 | | 39.5 |
| (K)IQDKEGIPPDQQR(L) | 508.60 | 1522.77 | | 39.5 |
| (K)IQDKEGIPPDQQR(L) | 762.39 | 1522.77 | | 39.7 |
| (R)LIFAG**K**QLEDGR(T) | 730.90 | 1459.78 | Ubiquitination (+114) | 38.3 |
| (R)LIFAG**K**QLEDGR(T) | 487.60 | 1459.78 | Ubiquitination (+114) | 38.4 |
| (R)TLSDYNIQK(E) | 541.28 | 1080.54 | | 37.8 |
| (K)ESTLHLVLR(L) | 534.31 | 1066.61 | | 31.3 |

**LEGEND TO SUPPLEMENTARY FIGURE**

a) Whole cell extracts were prepared from HeLa cells transfected with expression vectors for HA-UBQ, ELK-1-His, Hand1-His and FBXO25, as indicated. Cells were harvested after 48 h or treated with MG132 (20 µM) 42 h post-transfection and harvested after a further 6 h, as indicated, and subjected to IMAC under denaturing conditions. Isolated proteins were separated by gradient SDS-PAGE and analysed by immunoblotting with the antibodies indicated. The arrow indicates bands corresponding to ectopically expressed FBXO25. This experiment was performed twice.

b) Whole cell extracts were prepared from HEK293T cells transfected with an expression vector for Hand1-His or empty vector and subjected to IMAC under denaturing conditions. Isolated proteins were separated by gradient SDS-PAGE and analysed by immunoblotting with the antibodies indicated. NB, Hand1 is modified with endogenous ubiquitin.

c) Spectral counts of phosphate-modified Hand1 peptides (+80) detected in parallel with ubiquitin-modified peptides [Figure 3f] during MS analysis of sample shown in lane 2 of (b). Phosphorylation of T107 and S109 has been observed previously [Martindill, D.M., Risebro, C.A., Smart, N., Franco-Viseras Mdel, M., Rosario, C.O., Swallow, C.J., Dennis, J.W. and Riley, P.R. (2007) Nucleolar release of Hand1 acts as a molecular switch to determine cell fate. Nature Cell Biol. 9:1131-41].

## Supplementary Figure

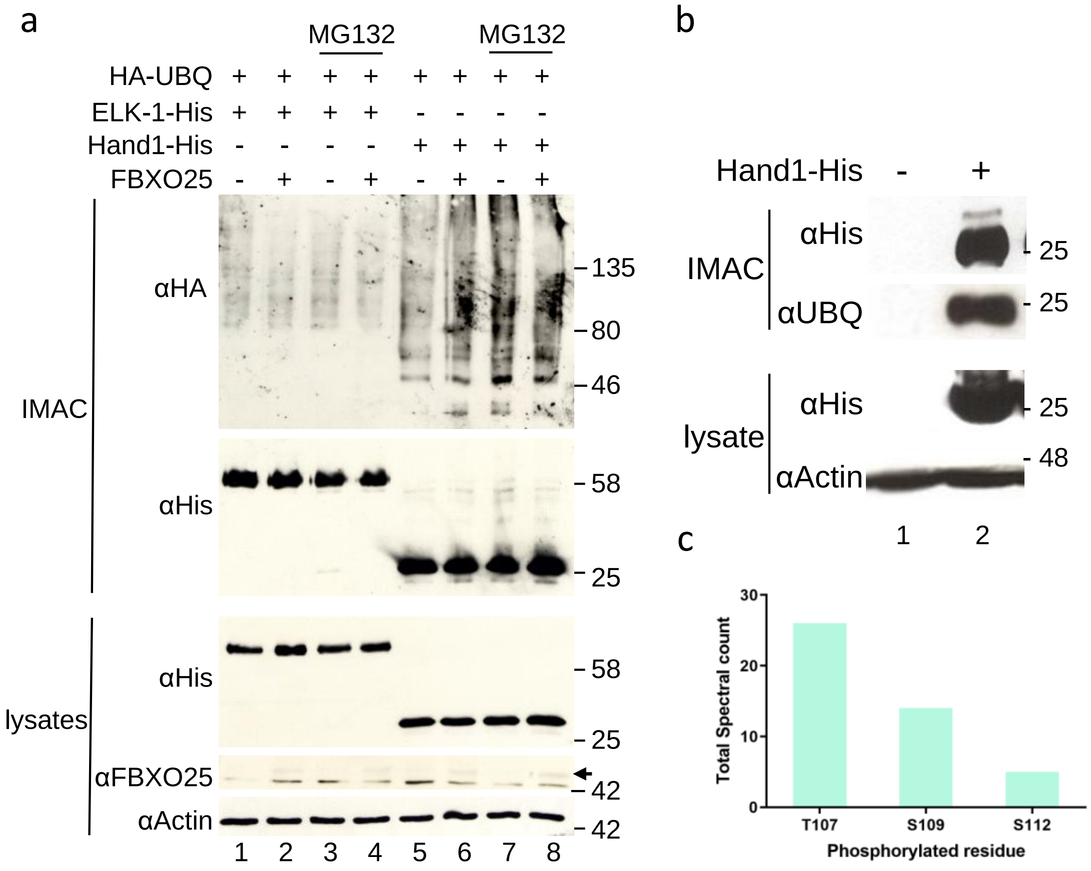

Supplement: Supplementary Information [file mmc1.pdf]
